# Antimicrobial effects of essential oil from *Origanum vulgare* in combination with conventional antibiotics against *Staphylococcus aureus*

**DOI:** 10.3389/fcimb.2025.1684624

**Published:** 2025-10-23

**Authors:** Elisabetta De Rose, Patrizia D’Aquila, Giada Sena, Annamaria Perrotta, Giuseppe Passarino, Dina Bellizzi

**Affiliations:** Department of Biology, Ecology and Earth Sciences, University of Calabria, Rende, Italy

**Keywords:** essential oil, *Origanum vulgare*, *Staphylococcus aureus*, minimum inhibitory concentration (MIC), antibiotics, biofilm, quorum sensing, DNA methylation

## Abstract

**Background:**

Antimicrobial resistance is emerging as a significant threat to public health, prompting the search for novel natural molecules, such as Essential Oils (EOs), that can affect, alone or in combination with conventional antibiotics, growth and various biological activities in microorganisms.

**Methods:**

First, the effects of ten essential oils extracted from aromatic plants grown in Calabria (Southern Italy) and seven conventional antibiotics against *Staphylococcus aureus* cells were studied individually, determining the Minimum Inhibitory Concentrations (MICs) through broth microdilutions. Subsequently, limited to *Origanum vulgare* EO (OEO) only, the compounds were evaluated in combination through checkerboard and time kill assays. ZIP synergy scores and Fractional Inhibitory Concentrations Indexes (FIC_I_) were calculated to determine the interactive effects of the combinations. At 0.5 x MIC concentration values of OEO-antibiotic combinations, the biofilm and the expression of genes involved in the Quorum Sensing (QS) process were determined by the crystal violet method and quantitative real-time PCR reactions, respectively. At the same concentrations, adenine and cytosine methylation levels were quantified through ELISA.

**Results:**

The results showed that *S. aureus* was highly sensitive only to OEO, in which a small MIC value was noticed (0.312 mg/mL). Synergistic effects were observed when combining OEO and ampicillin, gentamicin, tetracycline, and tobramycin, resulting in reductions of antibiotic MICs. An inhibition of biofilm formation and a general down-regulation of the expression of *agrA*, *hld*, *RNAIII*, and *rot* genes were observed. Similarly, up- and down-methylation of cytosines and adenines, respectively, compared to antibiotics alone was noticed.

**Conclusions:**

Taken together, our observations provide evidence on the role of the OEO-antibiotic combinations in enhancing the action of antibiotics on the growth and suggest that these combinations could influence biological processes such as biofilm formation, QS, and epigenetic changes.

## Introduction

Antimicrobial resistance (AMR) is rapidly becoming a major global health concern, accounting for approximately 9% of global deaths due to critical challenges in the management and eradication of an ever-increasing range of infections caused by bacteria, fungi, parasites, and viruses (Salam et al., 2023; Tang et al., 2023). In this context, *Staphylococcus aureus* stands out as one of the prevalent bacterial pathogens due to its great adaptability and increasing ability to develop or acquire resistance traits to antibiotic drugs over time, to the point of being included in the Bacterial Priority Pathogens List (BPPL) by the World Health Organization (Sati et al., 2025; Touaitia et al., 2025). *S. aureus* is a Gram-positive human commensal microorganism that, under certain conditions, can become an opportunistic pathogen capable of causing infections ranging from superficial skin to life-threatening systemic diseases, including bacteremia, infective endocarditis, pulmonary infections, meningitis, and toxic shock syndrome, especially when the host’s immune system is compromised or the bacteria breach protective barriers (Cheung et al., 2021). This is mainly due to the complex regulatory network of the Quorum Sensing (QS) system which manages virulence factors leading to toxin production, adhesion, and biofilm formation, allowing the pathogen to thrive in different environmental conditions (Yamazaki et al., 2024; Touaitia et al., 2025). What is more, mainly through genetic changes, *S. aureus* may develop resistance against a variety of clinically relevant antibiotics to which it was previously sensitive (Brdová et al., 2024; Shao et al., 2025). This poses an ever-increasing challenge not only in the development/research of new antibiotics but also in the exploration and optimization of alternative/combinatorial therapeutic approaches to overcome the limitations of conventional antibiotics and ensure more effective treatments for life-threatening *S. aureus* infections.

In this frame, interesting results come from natural bioactive compounds which exhibit a multitude of biological activities against bacteria (Arrigoni et al., 2024; Hamad et al., 2024). More particularly, essential oils (EOs), concentrated mixtures of volatile, hydrophobic and liposoluble compounds obtained from various parts of aromatic plants, have drawn considerable attention due to their chemically diverse composition rich in terpenes, terpenoids, and phenolic compounds which are responsible for antimicrobial properties against a wide range of bacteria, including antibiotic-resistant strains. Particularly, depending on the microbial strain and their composition, EOs may primarily act at the membrane level by modifying the cell wall biogenesis and membrane hydrophobicity and polarity, resulting in the destabilization of the cell membrane potential and the breakdown of membrane integrity (Swamy et al., 2016; Tariq et al., 2019; Bacińska et al., 2025; Ma et al., 2025a). In addition, the interaction between EO components and cell wall receptors leads to a dysregulation of vital bacterial metabolic pathways as well as the disruption of the signalling network of cell-to-cell communication, thus inhibiting Quorum Sensing and biofilm formation which ultimately leads to cell growth arrest and/or bacterial death (Gonçalves et al., 2023; D’Aquila et al., 2024; Maggio et al., 2025). Being a mixture of bioactive molecules, each acting on several targets, on the one hand this makes EOs effective against a variety of microorganisms, and on the other, the risk of inducing bacterial resistance is significantly reduced.

The most widely studied OE for its antimicrobial properties is certainly that obtained from *Origanum vulgare.* Mainly due to its high content of carvacrol and thymol, oregano essential oil (OEO) has shown particularly strong antibacterial activity against Gram-positive and Gram-negative and even multidrug-resistant bacterial and fungal microorganisms, including *S. aureus, Bacillus subtilis, Enterococcus faecalis, Escherichia coli, Pseudomonas aeruginosa*, and *Candida albicans*, exhibiting inhibitory effects on microbial growth, biofilm formation, swarming motility, and QS signaling (Fikry et al., 2019; Soltani et al., 2021; Merghni et al., 2022; D’Aquila et al., 2022, D’Aquila et al., 2023; Nurzyńska-Wierdak and Walasek-Janusz, 2025). What is more, a body of evidence reports that EOs can enhance the effectiveness of traditional antibiotics for improved therapeutic efficacy, overcoming/increasing the efficacy of clinical antimicrobials that have become ineffective due to the antimicrobial resistance issue as well as avoiding the undesirable toxic effects by lowering the dose of the antimicrobial treatment (Langeveld et al., 2014; Liu et al., 2023; Zhao et al., 2024; Ma et al., 2025a; Jilani et al., 2025). For instance, the antibiotic-potentiating effectiveness of a variety of EO, such as *Origanum compactum*, *Origanum elongatum, O. vulgare*, *Rosmarinus officinalis L, Thymus broussonnetii, Thymus pallidus* to combat planktonic cells, biofilms, and efflux pump activities were recently demonstrated in several bacterial and fungal strains, also including highly prioritized multidrug-resistant strain of *E. coli*, *E. faecalis*, *Klebsiella pneumoniae*, *P. aeruginosa*, *S. aureus, Salmonella enterica* and *Acinetobacter baumannii* (Amassmoud et al., 2023; Khashei et al., 2025; Drioiche et al., 2024; Sena et al., 2024).

Accordingly, the purpose of this study is to investigate the potential impact of Essential Oils extracted from ten aromatic plants in combination with seven commercial antibiotics against *S. aureus* growth, biofilm formation, Quorum Sensing regulation, as well as epigenetic modifications.

## Materials and methods

### Bacterial strains and growth conditions

This study was carried out on *S. aureus* strain (Merck NCTC 6571 Lenticule^®^ discs) kept frozen in stock cultures at −80°C in cryovials. Bacterial cells were cultured in Luria-Bertani medium containing 10 g of Bacto-Tryptone, 5 g of yeast extract, and 0.5 g of sodium chloride at 37°C under gentle agitation.

### EOs and antibiotics

Ten EOs were obtained from a commercial producer located in Calabria (Italy). They were extracted from the following plants: *Clinopodium nepeta L*. (Kuntze, Carl Ernst Otto), *Citrus bergamia* (Risso, Joseph Antoine & Poiteau, Pierre Antoine), *Citrus limon L.* (Osbeck, Pehr), *Citrus reticulata* (Blanco, Francisco Manuel), *Foeniculum vulgare* subsp. *piperitum*, *Laurus nobilis L., Myrtus communis L., Origanum vulgare L. subsp. viridulum* (Martrin-Donos, Julien Victor) Nyman, Carl Frederik, *Salvia officinalis L*., and *Salvia rosmarinus* (Spenn, Fridolin Carl). In particular, the fruit peel of *C. bergamia* and *C. limon*, the flower, leaf, and terminal branches of *C. nepeta*, *F. vulgare*, *M. communis*, *O. vulgare*, *S. officinalis*, and *S. Rosmarinus*, and only the leaf and terminal branches of *C. reticulata* and *L. nobilis* were used. The extraction procedure and composition analysis were carried out as described in D’Aquila et al. (2023). Since EOs are highly lipophilic organic mixtures, we dissolved them in 2% DMSO in growth medium in a working solution of 10 mg EO/mL.

Ampicillin (AMP), ciprofloxacin (CIP), erythromycin (ERY), gentamicin (GEN), levofloxacin (LVX), tetracycline (TET), and tobramycin (TOB) (Sigma-Aldrich, St Louis, MO, USA) were analyzed in the study. Working solutions of all antibiotics were prepared at a final concentration of 500 μg/mL.

### Determination of minimum inhibitory concentration of the EOs and antibiotics

The Minimum Inhibitory Concentration (MIC) of the EOs and antibiotics was determined by the broth microdilution method carried out in multi-well microplate. 10 μL of bacterial suspension from overnight cultures of *S. aureus* were inoculated into 96-well plates (~5 x 10^4^ CFU/well) containing 1:2 serial dilutions of the working solution of EOs or antibiotics at final volume of 150 μL (final DMSO concentrations from 1% to 0.00098%). Microplates were incubated for 22 h at 37°C. In all experiments, positive (medium with cells) and negative (medium without cells) control free of EOs and antibiotics were also analyzed to verify the appropriateness of microbial growth and sterility, respectively. In addition, two further controls were represented by the cell-free medium in the presence of each EO to discern the turbidity background, as well as by the medium-containing cells in the presence of 2% DMSO free of EOs, to confirm that this concentration has no effect on bacterial growth. Turbidity measurement was performed at 600 nm in a spectrophotometer. MIC values were determined as the lowest concentrations of EO and antibiotics corresponding to values of optical density (OD) comparable to those of the cell-free medium. Each experiment was carried out in triplicate, with three independent repetitions.

### Checkerboard assay

The interaction between the EO extracted from oregano (OEO) and the different antibiotics was analyzed using the checkerboard assay, as previously described.

10 μL of bacterial suspension from overnight cultures of *S. aureus* were inoculated into 96-well plates (~5 x 10^4^ CFU/well) containing twofold dilutions of OEO and antibiotics at concentrations ranging from five values ​​below and one above the MIC values at final volume of 150 μL (final DMSO concentrations from 0.125% to 0.0019%). After 22 h of incubation at 37°C, the OD_600_ values were measured using a microplate reader to determine the new MIC values for the mixtures and to calculate vitality percentages as follows: (ODuntreated - ODtreated/ODuntreated) x 100. Each experiment was carried out in triplicate.

The interactions between OEO and antibiotics combinations over the entire range of concentrations were analyzed through the free web-based SynergyFinder software (https://synergyfinder.org/) that exploits the Zero Interaction Potency (ZIP) calculation model which examines the drug interaction relationships by comparing the change in the potency of the dose-response curves between individual drugs and their combinations (Zheng et al., 2022).

The ZIP scores of the OEO and antibiotic combinations were interpreted as follows: synergism (>10), additive (<10 and >–10), and antagonistic (<–10) (Ianevski et al., 2017; 2020).

The lowest concentrations of OEO and antibiotic that showed an inhibition greater than 99% were used to calculate the Fractional Inhibitor Concentration Index (FIC_I_) through the following formula: FIC_I_ = FIC_OEO_ + FIC_antibiotic_, with FIC_OEO_ = (MIC of OEO in combination with antibiotic)/(MIC of OEO) and FIC antibiotic = (MIC of antibiotic in combination with OEO)/(MIC of antibiotic alone). The results were interpreted according to Fratini et al. (2017) as follow: total synergism when FIC_I_ < 1; FIC_I_ =1 indicates additivity; 1 < FICI ≤ 2 no interaction; and FICI > 2 antagonism. The MIC gain of the antibiotics was calculated according to the following formula: MIC gain = MICantibiotic/MIC antibiotic+OEO inferred by considering the lowest sub-MIC concentration values ​​at which an inhibition in bacterial growth of >99% was observed.

### Time-kill tests

Time-kill experiments were performed in 96-well plates containing combinations of OEO and antibiotics that elicit a synergistic effect on checkboard dilution analysis (0.16 mg/mL of the OEO and 0.063 μg/mL of AMP, 0.08 mg/mL of the OEO and 0.25 μg/mL of GEN, 0.16 mg/mL of the OEO and 0.03125 μg/mL of TET, and 0.08 mg/mL of the OEO and 1 μg/mL of TOB). The effect of OEO and antibiotics alone was also monitored. Briefly, bacterial cells were placed on microtiter plates (∼5 × 10^4^ CFU/well), and incubated at 37°C. At time intervals of 0, 2, 4, 6, and 24 h, cultures were serially diluted at 1:10 in sterile saline solution and 10 μL were plated onto LB agar plates and incubated for 24 h at 37°C. Then, the viable colonies were counted and represented as log_10_ (CFU/mL) The combination is considered synergic when it causes a ≥2log_10_ reduction in CFU/mL with respect antibiotic alone at 24 hours (NCCLS, 1999).

### Biofilm assay

Combinations of OEO-antibiotics were assessed for their potential on bacterial biofilm. 10 μL of suspension from overnight cultures of *S. aureus* were inoculated into 96-well plates (~5 x 10^4^ CFU/well) containing 0.5x MIC ​​of the synergic OEO-antibiotic combinations at final volume of 150 μL. After 22 h of incubation at 37°C, the planktonic cells were removed, and adherent cells were fixed with 150 μL of methanol and then stained with 150 μL of crystal violet solution 0.2%. After 5 min, the excess of the stain was removed by three repeated washes with 150 μL of isotonic saline solution (0.9% sodium chloride). 150 μL of acetic acid at a concentration of 33% was added to each well and the optical density at 570 nm was determined spectrophotometrically. As controls of experimental procedure, wells containing only the inoculated bacteria and the cell-free growth medium supplemented with OEO, respectively, were analyzed. Each experiment was carried out in triplicate. Effects of treatment on biofilm are expressed as Fold Change in the optical density values of samples treated with OEO-antibiotic combinations compared to that of the antibiotic alone.

### DNA and RNA extraction

50 μL of bacterial suspension from overnight cultures of *S. aureus* were inoculated into tubes (~5 x 10^7^ CFU/tube) containing 0.5x MIC ​​of the synergic OEO-antibiotic combinations at final volume of 3 mL. After 22 h of incubation at 37°C, DNA and total RNA were be extracted by using the “ZymoBIOMICS DNA/RNA Miniprep Kit” (Zymo Research). Briefly, cell culture was collected and centrifuged for 10 minutes. Isolated pellets were resuspended in 750 µl DNA/RNA Shield, transferred to a ZR BashingBead Lysis Tube and vortexed for 5 minutes. After centrifugation at 12,000 x g for 30 seconds, 400 µl of DNA/RNA Lysis Buffer was added to the supernatant, and the solution was transferred to a Spin-Away™ Filter and centrifuged at 12,000 x g for 30 seconds. After adding an equal volume of 70% ethanol, the eluate, containing the RNA fraction, was transferred to a ZymoSpin™ IIICG Column and centrifuged at 12,000 x g for 30 seconds, while 400 µl of DNA/RNA Prep Buffer were transferred into the Spin-Away™ Filter column, containing the DNA. After centrifugation at 12,000 x g for 30 seconds, two consecutive washes were performed in the presence of DNA/RNA Wash Buffer. 100 µl of ZymoBIOMICS™ DNase/RNase-Free water was transferred to the matrices of the two columns which, after incubation for 5 minutes at room temperature, were centrifuged. The DNA and RNA present in the two eluates were finally transferred onto previously reconstituted Zymo-Spin™ III-HRC Filter columns and isolated following a centrifugation at 16,000 x g for 3 minutes. The concentration and purity of the nucleic acids were determined spectrophotometrically using the absorbance ratio of 260/280 nm.

### Expression profiles of genes encoding enzymes involved in quorum sensing processes

The reverse transcriptase-PCR (RT-PCR) reaction was performed using the “High-Capacity cDNA Reverse Transcription Kit” (Thermofisher). Briefly, in a final volume of 20 μl, 250 ng of total RNA were mixed with a reaction mixture consisting of RT Buffer 1X, dNTP mix 4 mM, RT Random Primers 1X, MultiScribe™ Reverse Transcriptase 50 U, and RNase Inhibitor 20 U. Then, the samples were incubated at 25°C for 10 minutes, then at 37°C for 120 minutes and, successively, at 85°C for 5 minutes to inactivate the reverse transcriptase.

The obtained cDNAs were then used as template in Real-Time PCR reactions performed in a QuantStudio3 Real-Time PCR system (Thermo Fisher Scientific). Forward and reverse primers specific for the genes involved in the Quorum Sensing process in *S. aureus* are reported in **Table 1** (Mootz et al., 2015; Sharifi et al., 2021; Martínez et al., 2023).

**Table 1:** Nucleotide sequence (5’→3’) of primers used in the quantification of mRNA levels of *agrA, hld, RNAIII, and rot* genes encoding components of the QS system in *S. aureus*, as well as of the 16S ribosomal gene (16S RNA), used as an internal control.

Gene	Primer forward	Primer reverse
*agrA*	CAACCACAAGTTGTTAAAGCAG	TCGTTGTTTGCTTCAGTGATTC
*hld*	ATTTGTTCACTGTGTCGATAATCC	GGAGTGATTTCAATGGCACAAG
*RNAIII*	CATGGTTATTAAGTTGGGATGGC	GAAGGAGTGATTTCAATGGCACA
*rot*	AAGAGCGTCCTGTTGACGAT	TTTGCATTGCTGTTGCTCTA
*16SRNA*	AGCCGACCTGAGAGGGTGA	TCTGGACCGTGTCTCAGTTCC

Primer efficiencies were determined using five 10-fold serial dilutions of cDNA, and all primer pairs (*agrA, hld, RNAIII, rot,* and 16S rRNA) showed amplification efficiencies within the acceptable range (98.3% to 99.66%) and high linearity (0.985≥R^2^ ≤ 1).

The final PCR mixture (15 µL) contained 1 µL of cDNA, SensiFAST SYBR Hi-ROX Mix 1X (Bioline, London, UK) and 0.2 µM of each primer. The thermal profile used for the reaction included a 2-minutes heat activation of the enzyme at 95°C, followed by 35 cycles of denaturation at 95°C for 15 seconds and annealing/extension at 60°C for 60 seconds, followed by melt analysis ramping at 60–95°C with a melt peak level of 10%. The presence of single melting peaks was verified. All measurements were taken in the log phase of amplification. Negative controls (in which water instead of cDNA was added) were also run in each plate. Real-time PCR data were analyzed by Diomni™ Design and Analysis (RUO)3.0.2 (Applied Biosystem) through the comparative Ct method, according to which the 2^dCt values of each target gene were normalized to that of 16S ribosomal gene (16S RNA). Then, the relative quantification of gene expression was determined by using the values determined in the cells subjected to the antibiotic alone as Reference Values.

### Quantification of global N6-methyladenosine and 5-methylcytosine levels

Global DNA methylation levels of N6-methyladenosines (m6A) and 5-methylcytosines (5mC) were determined by using the MethylFlash m6A DNA Methylation ELISA Kit (Epigentek, Farmingdale, Nassau County, NY, USA) and the MethylFlash Global DNA Methylation (5mC) ELISA Easy Kit, respectively, following the manufacturer’s instructions. Shortly, the methylated fraction of bacterial genomic DNA, through ELISA-like reactions, was recognized by the m6A or 5mC antibodies and quantified in a microplate spectrophotometer by reading the absorbance at 450 nm. In each experiment, the percentage of m6A and 5mC was calculated using the second-order regression equation of a standard curve that was constructed by mixing equivalent molar concentrations at different ratios of full unmethylated and methylated control DNA. Each sample was analyzed in triplicate. The methylation values of DNA samples extracted from cells treated with antibiotic alone were used as reference values (Relative Quantification, RQ) for the corresponding samples treated with combinations of OEO and antibiotics.

### Statistical analysis

Statistical analyses were performed using SPSS 28.0 statistical software (SPSS Inc., Chicago, IL, USA). Kruskal–Wallis one-way analysis of variance and Mann-Whitney tests were adopted. Significance level was defined as p ≤ 0.05.

## Results

### Antimicrobial activity of the EOs

In the present study, ten EOs belonging to *Apiaceae* (*F. vulgare* subsp. *Piperitum*), *Lamiaceae* (*C. nepeta*, *O. vulgare L*. subsp. *Viridulum*, *S. officinalis* L., *S. rosmarinus*), *Lauraceae* (*L. nobilis L.*), *Myrtaceae* (*M. communis L.*) and *Rutaceae* (*C. bergamia*, *C. limon*, *C. reticulata*) family plants were analyzed. Plant family characteristics and a complete description of all EOs are shown in **Supplementary Table S1** and **Supplementary Table S2** (Sena et al., 2024).

The results from the broth microdilution assay in terms of MIC are reported in **Table 2**.

**Table 2:** MIC values of the essential oils (mg/mL) and the antibiotics (µg/mL) examined in the study.

Treatments		MIC values
Essential oils	*C. nepeta*	> 5
	*C. bergamia*	> 5
	*C. limon*	> 5
	*C. reticulata*	> 5
	*F. vulgare*	> 5
	*L. nobilis*	> 5
	*M. communis*	> 5
	*O. vulgare*	0.312
	*S. officinalis*	> 5
	*S. rosmarinus*	> 5
Antibiotics	AMP	0.500
	CIP	0.125
	ERY	0.250
	GEN	2
	LVX	0.250
	TET	1
	TOB	2

The analysis revealed that the only EO that showed significant antibacterial activity was the one extracted from *O. vulgare*, as deduced from the MIC values ​​equal to 0.312 mg/mL. Under our laboratory conditions, the remaining EOs did not show antibacterial activity, so they were not further investigated.

Regarding antibiotics, the MIC values ​​observed are in line with those reported in the MIC European Committee on Antimicrobial Susceptibility Testing (EUCAST) database (https://www.eucast.org”) in which MIC distributions for individual microorganisms are reported.

The MIC values of OEO and antibiotics were then used to evaluate the effects against bacteria when they were analyzed in combination with each other.

### Effects of the combination of the OEO and antibiotics against *S. aureus*

Combinations of AMP, CIP, ERY, GEN, LVX, TET, and TOB with OEO against *S. aureus* were tested through checkerboard assays, and ZIP model was used to analyze the OEO-antibiotic interactions over the entire range of concentrations.

**Figure 1:**
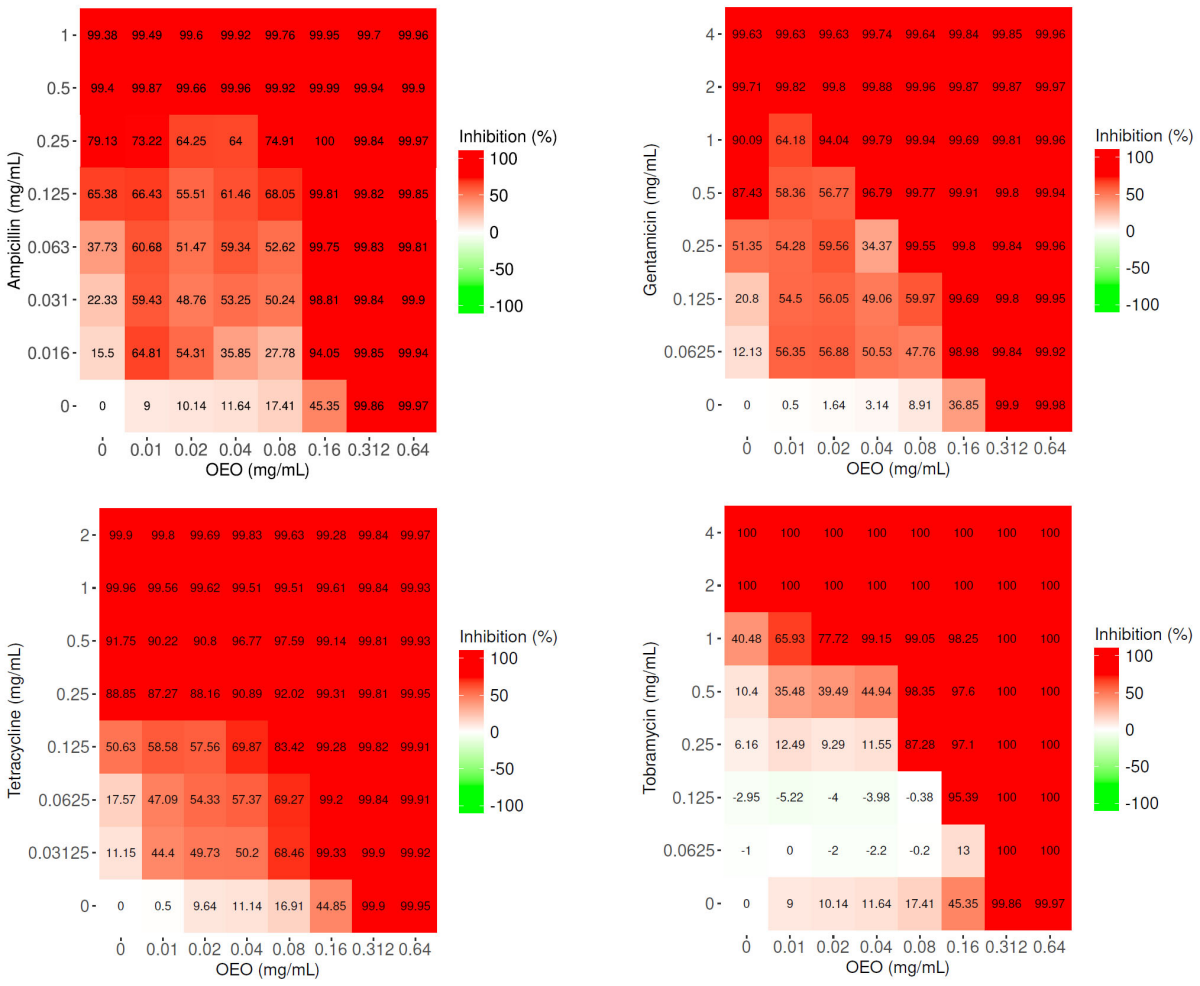
Matrices of interaction relative to the combinations of OEO (mg/mL) with ampicillin, gentamicin, tetracycline, and tobramycin (μg/mL). Values represent the average of three independent experiments.

The resulting matrices of interaction show that only the combinations of OEO with AMP, GEN, TET, and TOB increase antibacterial activity against *S. aureus* when compared to OEO or antibiotic alone (p<0.001) (**Figure 1**; **Supplementary Table S3**).

If we consider the lowest sub-MIC concentration values of OEO and antibiotics ​​at which an inhibition in bacterial growth of >99% occurs, the association of the OEO with AMP determined a gain in the antibiotic MIC of about 8-fold (0.16 mg/mL of the OEO and 0.063 μg/mL of the AMP), with GEN of 8-fold (0.08 mg/mL of the OEO and 0.25 μg/mL of the GEN), and with TOB of 2-fold (0.08 mg/mL of the OEO and 1 μg/mL of the TOB). Notable, a reduction of MIC value was appreciated, with a gain of up to 32-fold, in the case of TET (0.16 mg/mL of the OEO and 0.03125 μg/mL of the TET) (**Figure 1**).

**Figure 2:**
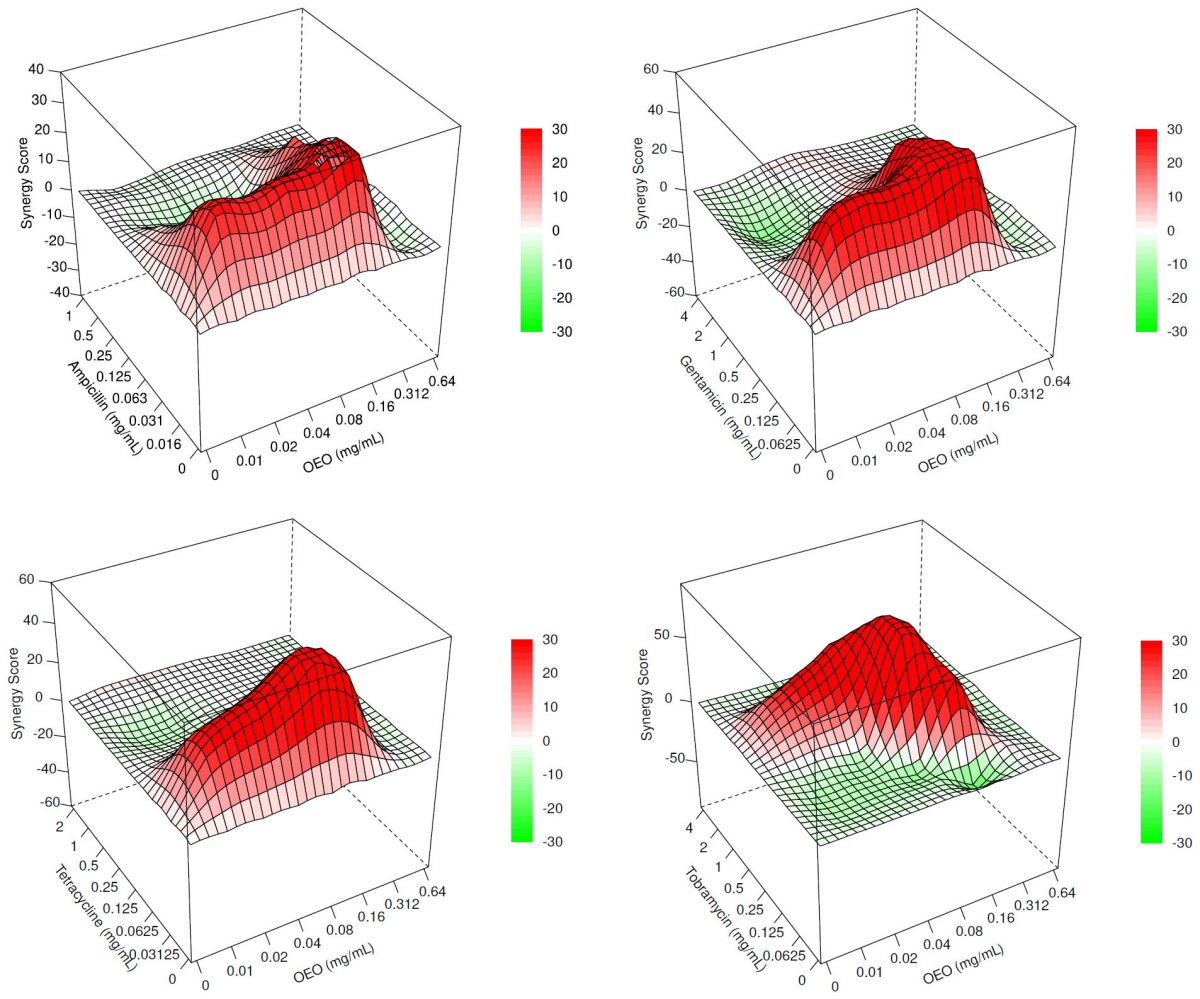
Surface plots representing the ZIP scores highlighting the interaction occurring between OEO and ampicillin, gentamicin, tetracycline, and tobramycin. Synergistic (>10), additive (<10 and 638 >–10), and antagonistic (<–10) effects.

The ZIP scores, graphically represented as surface plots in **Figure 2** and reported as data in **Supplementary Table S4**, highlight a synergism occurring between OEO and the four antibiotics. In particular, different combinations of OEO and antibiotics showed ZIP scores >10. Accordingly, these conditions of synergy were also confirmed by determining FIC_I_ values (**Supplementary Table S4**).

CIP, ERY, and LVX did not exhibit significant change of the antibacterial activity when tested with OEO at various concentrations and were, therefore, not investigated further.

### Time-kill curves

**Figure 3:**
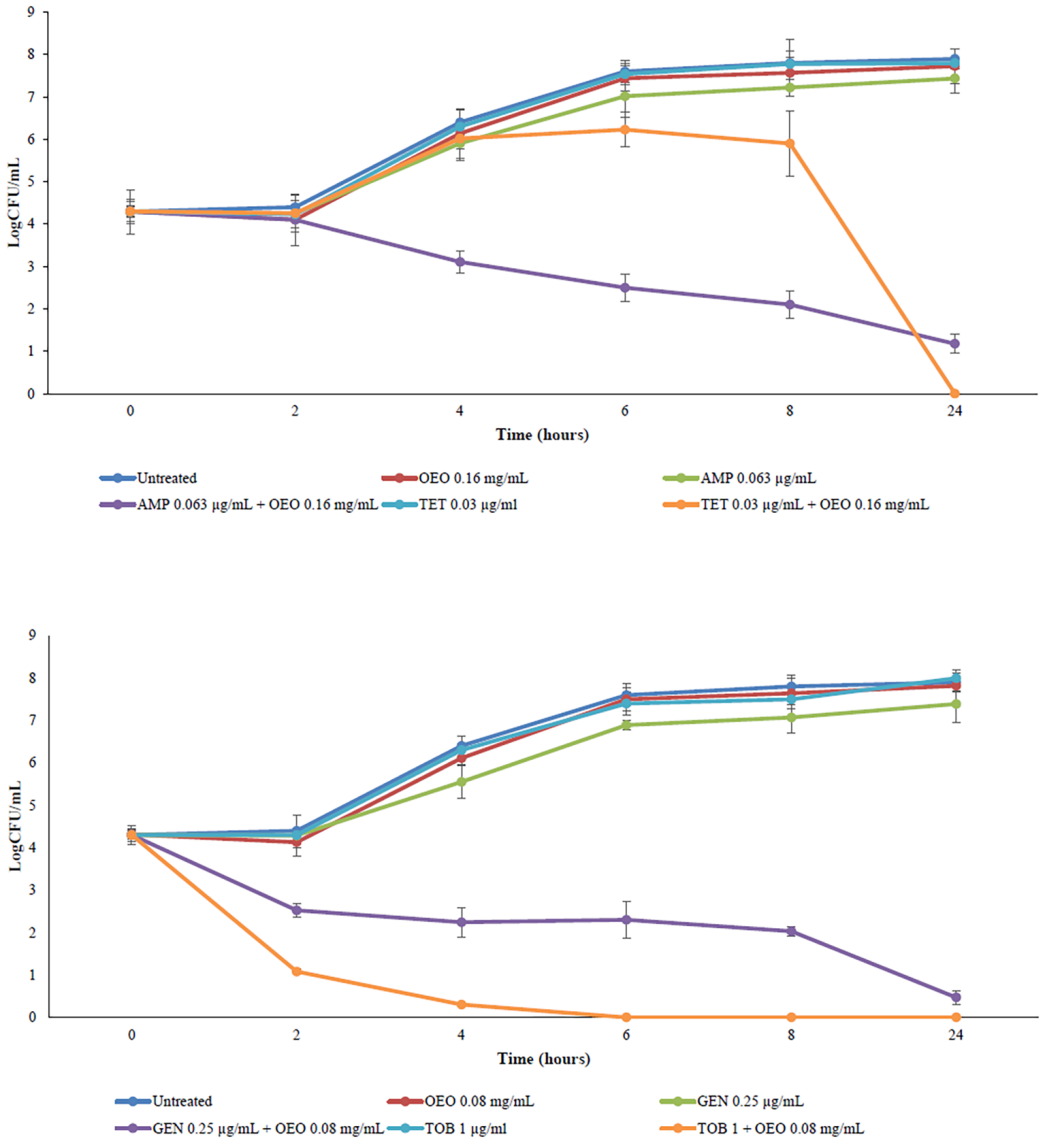
Time-kill curves of OEO combining with ampicillin, gentamicin, tetracycline, and tobramycin against *S. aureus*.

The best synergistic combinations from checkerboard assay (0.16 mg/mL of the OEO and 0.063 μg/mL of AMP, 0.08 mg/mL of the OEO and 0.25 μg/mL of GEN, 0.16 mg/mL of the OEO and 0.03125 μg/mL of TET, and 0.08 mg/mL of the OEO and 1 μg/mL of TOB) were evaluated through time-kill assay. We compared the bacterial killing kinetics of antibiotics and OEO antibiotic combinations. According to the checkerboard assay, all the OEO antibiotic combinations kill *S. aureus* as emerged by a decrease greater of 2log_10_ in CFU/mL cells within 24 hours. OEO combining with AMP showed the antibacterial activity of 2.8log_10_ in CFU/mL from 6 hours towards, combining with GEN of 2.5log_10_ in CFU/mL from 4 hours towards, with TOB of 4log_10_ in CFU/mL from 2 hours towards(**Figure 3**). These findings demonstrated that OEO combining antibiotics exhibit different efficacy over time depending on the specific antibiotic.

### Effects on biofilm induced by combinations of OEO with antibiotics

**Figure 4:**
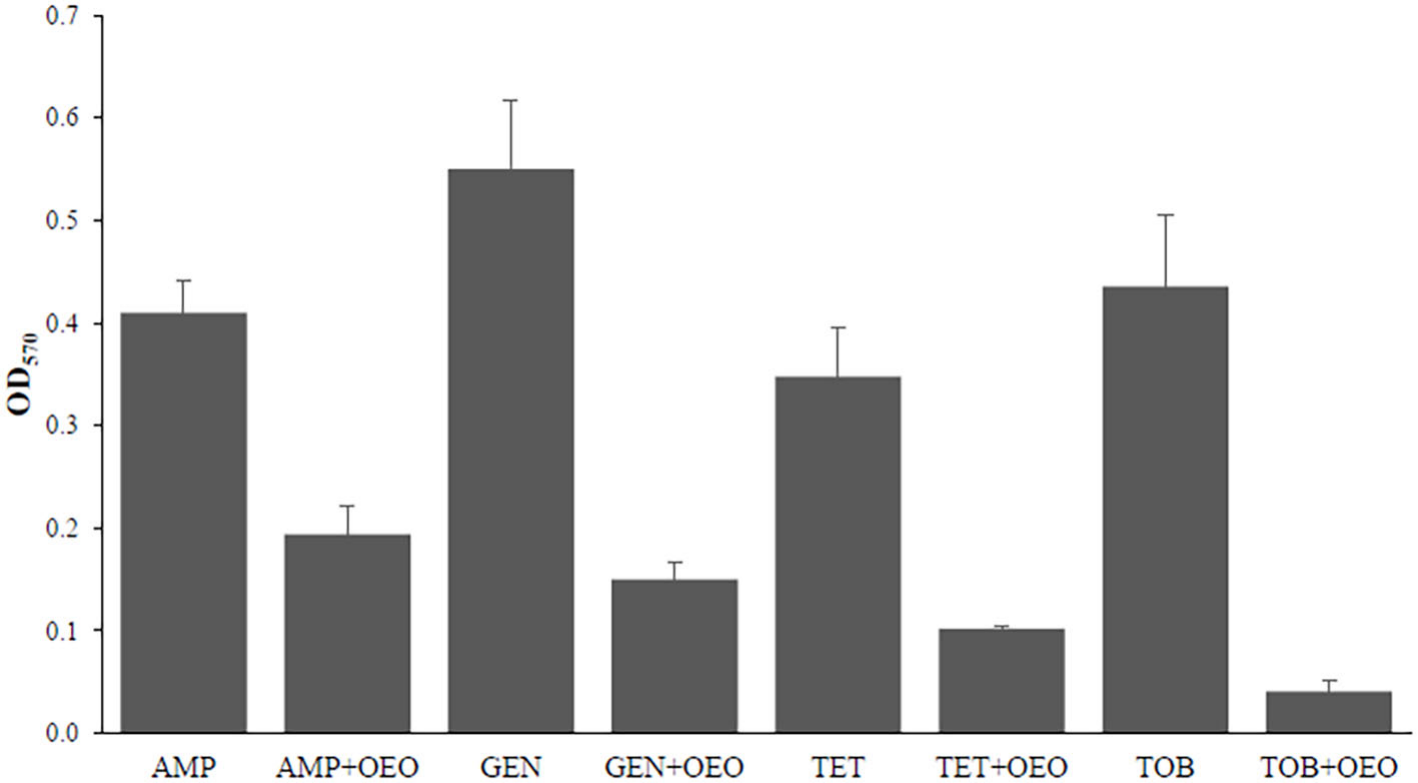
Effects induced by the combination of OEO and antibiotics on biofilm in *S. aureus*. Values are reported in terms of Fold Change determined using cells treated with the antibiotic alone as reference. Values represent the average of three independent experiments with a standard error of the mean. AMP, ampicillin; GEN, gentamicin; TET, tetracycline; TOB, tobramycin.

**Figure 4** reports the effects on biofilm inhibition in *S. aureus* exerted by combinations of OEO with antibiotics. All the combinations of the OEO with antibiotics were able to inhibit biofilm with inhibition percentages of 53% in the combination of OEO with ampicillin (p<0.01), 73% with gentamicin (p<0.001), 71% with tetracycline (p<0.001), and 91% with tobramycin (p<0.001)(**Supplementary Table S5**).

### Effects on quorum sensing induced by combinations of OEO with antibiotics

**Figure 5:**
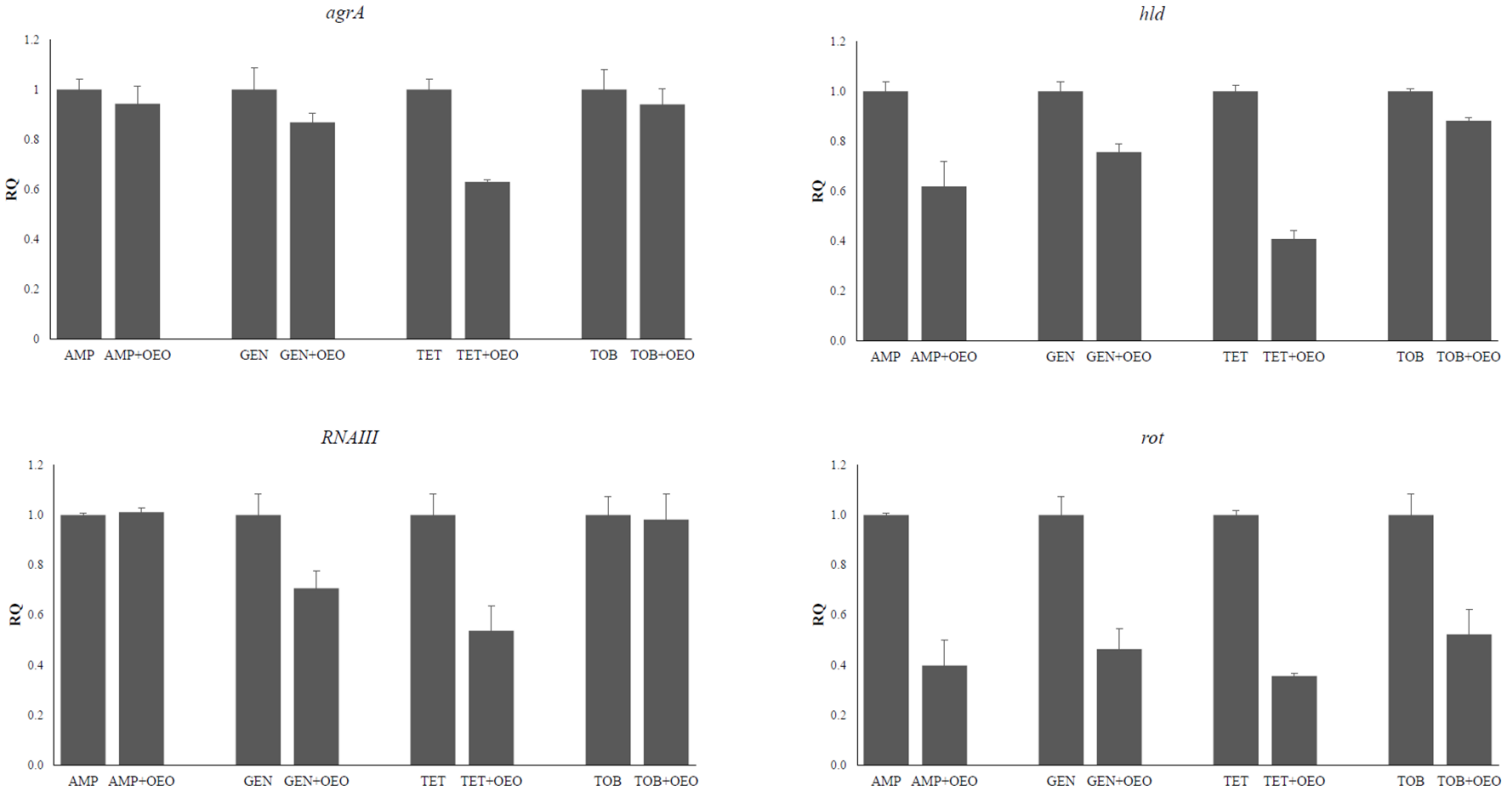
Effects induced by the combinations of OEO and antibiotics on the expression of *agra*, *hld*, *RNAIII*, and *rot* genes. Values are reported as Relative Quantification of gene expression determined by using the values determined in the cells subjected to the antibiotic alone as reference. Values represent the average of three independent experiments with a standard error of the mean. AMP, ampicillin; GEN, gentamicin; TET, tetracycline; TOB, tobramycin.

The expression levels of *agrA, hld, RNAIII, and rot* genes encoding components of the QS system in *S. aureus* were evaluated following combined treatment of cells with OEO and antibiotics. We can observe a more marked decrease in expression for the genes *agrA* at the combination of OEO with tetracycline (p<0.01), *hld* with ampicillin (p<0.001), gentamicin (p<0.001), tetracycline (p<0.001), and tobramicin (p<0.01), *RNAIII* with gentamicin (p<0.001), and tetracycline (p<0.001) and *rot* with all four antibiotics (p<0.001) (**Figure 5**; **Supplementary Table S6**). No significant changes in gene expression were observed in the other combinations.

### Effects of the synergism of OEO with antibiotics on global methylation profiles

**Figure 6:**
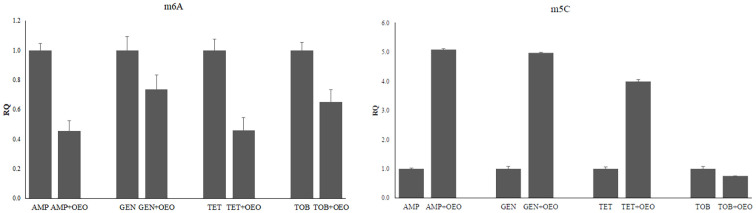
Effects induced by the combinations of OEO and antibiotics on methylation of adenines (m6A) and cytosines (m5C). Values are reported as Relative Quantification (RQ) determined by using cells treated with the antibiotic alone as reference. Values represent the average of three independent experiments with a standard error of the mean. AMP, ampicillin; GEN, gentamicin; TET, tetracycline; TOB, tobramycin.

The global methylation levels of adenine and cytosine residues were evaluated in DNA samples extracted from *S. aureus* cells and treated with OEO combined with antibiotics. Standard curves were generated by plotting the absorbance values for each combination versus percentage of m6A (detection range 0.02-0.2 ng) and 5-mC (detection range 0.1-5%) observing a logarithmic relationship with a correlation of 0.97 and 0.99, respectively, thus confirming the effectiveness of the experimental conditions (**Supplementary Figure S1**). By comparing global DNA methylation levels of adenines (m6A) quantified at the different combinations of OEO with antibiotics with those in presence of the antibiotic alone, we can observe significant hypo-methylation in all OEO and antibiotic combinations (p<0.01). As concerns 5mC levels, a hypermethylation is evident in the combinations of OEO with ampicillin, gentamicin, and tetracycline meanwhile hypomethylation occurs when the cells are treated with OEO combined with tobramycin (P<0.01) (**Figure 6** and **Supplementary Table S7**).

## Discussion

The global increase in antimicrobial resistance is driving the urgent identification of alternative antimicrobial approaches to improve treatment of infectious diseases and health care outcomes. In this context, the recent discovery of plant-derived components with effective antibacterial properties suggests as they can serve, alone or in combination with other products as new and effective antibacterial therapies.

The present study explored the antibacterial properties of ten EOs extracted from aromatic plants (C*. nepeta*, *C. bergamia*, *C. limon*, *C. reticulata*, *F. vulgare, L. nobilis*, *M. communis*, *O. vulgare*, *S. officinalis*, and *S. rosmarinus*) against *S. aureus*, considering both its very high pathogenic potential and its strong ability to develop antibiotic resistance, making it an extremely problematic pathogen in clinical settings. Among the EOs analyzed, the one extracted from *O. vulgare* (OEO) showed the highest antimicrobial activity (MIC = 0.312 mg/mL), further confirming its role as effective antibacterial against a whole series of both antibiotic-resistant and non-resistant bacterial strains, such as *A. baumannii, E. faecalis, E. coli, K. pneumoniae, Listeria monocytogenes*, *P. aeruginosa, Salmonella enteritidis, Salmonella Typhimurium, Streptococcus mutans*, and *S. aureus* (Sakkas and Papadopoulou, 2017; Yuan et al., 2023; Tejada-Muñoz et al., 2024; Ezzariga et al., 2025; Tao et al., 2025). As reported in Saoudi et al. (2024), the mechanism of action of EOs is strictly dependent on the quality and quantity of their chemical compounds. In fact, their antibacterial activity does not result from a single mechanism, but rather from a cascade of reactions involving the entire bacterial cell. The remarkable efficacy of the OEO we analysed appears to be attributed to the presence of high percentages of thymol, terpinene, and p-cymene, which are the main components of the oil analyzed in this study. In particular, thymol disrupts bacterial cell membranes, causing leakage of cellular contents and ultimately leading to cell death. It can also inhibit efflux pumps which are involved in antibiotic resistance (Langeveld et al., 2014; Sakkas and Papadopoulou, 2017; Walsh et al., 2003).

Diversity in the antibacterial activity of the EO from *O. vulgare* is mainly ascribed to the abundance percentage of its principal constituents, which vary considerably depending on altitude, temperature, harvest season, and geographical location, and extraction methods (Piasecki et al., 2023; Tejada-Muñoz et al., 2024).

Few studies have reported that OEO can synergize with certain antibiotics, enhancing their effectiveness against bacteria of clinical importance and potentially reducing the required dosage of the antibiotics. For further expanding the data of synergistic effects, the interactions between the OEO and conventional antibiotics were assayed through checkerboard method. It emerged that the combination of OEO with ampicillin, gentamicin, tetracycline, and tobramycin significantly enhance the antibacterial activity of the antibiotics against *S. aureus* when compared to antibiotic or OEO alone, as demonstrated by the application of effective models describing the synergism between different compounds and by the low FIC_I_ values. Depending to the antibiotic, these combinations decrease antibiotic dosage able to inhibit *S. aureus* growth from a minimum of 2 to a maximum of 32 folds.

Results obtained from time-kill kinetics assays provide more complementary evidence for the observed synergy effect of OEO antibiotic combination generated by our checkerboard assay since the combinations were more effective against *S. aureus* than the antibiotic alone, displaying an overtime bactericidal action antibiotic-dependent.

As reported by Drioiche et al. (2024), these synergistic effects could result from their ability to target the same bacterial proteins or facilitate access to target sites, as suggested by molecular docking simulations. Results we observed are in line with some studies, such as that of Xiao et al. (2020) who conducted a large-scale study analyzing the activity of 139 essential oils, demonstrating that, when combined with some currently recommended antibiotics for *S. aureus* infections, OEO showed a positive enhancement effect in increasing the activity of some antibiotics (Rosato et al., 2010; Amassmoud et al., 2023; Sena et al., 2024; Drioiche et al., 2024; Khashei et al., 2025). What is more, our results appear particularly interesting because they prove that the synergistic effect of OEO is antibiotic specific. Indeed, OEO is most effective when combined with β-lactam and tetracyclines antibiotics, less effective with aminoglycosides, and ineffective in combination with fluoroquinolones and macrolides. Furthermore, the reduction of the minimum dose necessary for each drug to exert its antibacterial effect is particularly relevant, if we consider the need to reduce not only the use of antibiotics, but also their possible toxic effects, such nephrotoxicity and ototoxicity, often associated with their high dosage and long-term exposure (Tyers and Wright, 2019; Coates et al., 2020). It is difficult to propose general antibacterial mechanisms of essential oil-antibiotic combinations owing to those specific biological properties of the OEO, which we mentioned earlier, and to distinct sensitivity of the microorganisms. However, as reported in literature, it seems that one of the most recognized mechanisms that could lead to this synergistic effect between OEO and intracellular-targeted antibiotics, such as gentamycin and tobramycin, is the destabilization of the bacterial membrane by oil components, which facilitates the penetration of the antibiotics towards the cytoplasm and better reaches their targets. With β-lactam and tetracycline antibiotics, the synergy could be due to the ability of certain oil components to inhibit bacterial resistance mechanisms such as efflux pumps that can pump antibiotics out of the bacterial cell before they can have effect. Lastly, combination of the OEO with β-lactams, which interfere with peptidoglycan formation, may have synergistic effects enhancing the disruption of bacterial cell walls (Aelenei et al., 2016; Saoudi et al., 2024).

Interestingly, the synergic OEO-antibiotic combinations exhibited antibiofilm activity since they are more effective at interfere with biofilm formation than either the OEO or antibiotic alone, as demonstrated by inhibition percentages of up to 91%. This activity could be attributed to the ability of OEO components to synergistically act with antibiotics in interfering with the various processes involved in biofilm formation, such as QS. This would seem to be confirmed by the downregulation we observed of *agrA, hld, RNAIII, and tot* genes encoding key components of the QS system in *S. aureus*. The *agr* locus encodes a two-component QS system that modulates the synthesis of the transcriptional regulator RNA III and the autoregulation of the agr system. The fact that OEO-antibiotic combinations influence the expression of the *RNA III* gene, significantly reducing its transcription, demonstrates its role in *S. aureus* biofilm formation. It is responsible for the post-transcriptional regulation of several virulence factors, which in turn influence the expression of cell surface-related proteins and extracellular toxins such as alpha-hemolysin (hla) and delta-hemolysin (hld) (Martínez et al., 2023). Negative regulation of the *hld* gene by the OEO-antibiotic combinations suggests that they affect not only hemolysin synthesis but also QS signal recognition proteins, causing inhibition on the expression of transcriptional regulators, toxin production, and biofilm formation.

As we have already reported in a previous study, combinations of EOs and antibiotics induce changes of global methylation levels of cytosines and adenines compared to EO or antibiotics alone. Results we obtained in the present study confirmed that overall, the OEO-antibiotic combinations induce a hypermethylation of cytosines and a downregulation of adenines even if these changes are specific for each antibiotic analyzed, thus shedding light on the molecular mechanisms through which the combinations exert their effects at the intracellular level. As we stated previously whether these changes are correlated to the inhibition or activation of gene expression even today remains unclear. One thing is certain: in different experimental conditions based on different antibiotics in different bacterial strains, epigenetic changes seem to recur in mediating the effects of the EO-antibiotic combinations in bacteria. Similarly, although a number of literature data report the enzymes involved in establishing and maintaining bacterial DNA methylation, it remains to be experimentally determined which of these enzymes are affected by oils and their combinations with antibiotics (Oliveira and Fang, 2021; Gao et al., 2023; Ma et al., 2025b).

Results we obtained in this study support the use of essential oils in combination with antibiotics as new treatment modalities to the bacterial infections opening the door for more research into the related field. If these data will be further confirmed, the adoption of novel therapeutic approaches might be used not exclusively against pathogenic bacteria, but primarily against multidrug-resistant bacteria, which are progressively increasing and against which conventional antibiotics appear to be ineffective. We are aware that, despite the promising results obtained so far in basic research which are confirmed in a plethora of works, challenges remain, such as the stability, selectivity, and bioavailability of these natural products in the human body and any potential adverse reactions. These factors need to be further explored to provide sufficient evidence for their effective application in the clinical field.

## Data Availability

The original contributions presented in the study are included in the article/**Supplementary Material**. Further inquiries can be directed to the corresponding author.

## Supplementary material

Supplementary Figure 1 Representative standard curves generated with m6A and m5C standard controls.
