# Supplementary figures and images for: Antimicrobial effects of essential oil from Origanum vulgare in combination with conventional antibiotics against Staphylococcus aureus
Source: Front Cell Infect Microbiol. 2025 Oct 23;15:1684624. doi: 10.3389/fcimb.2025.1684624 (PMC12588934; doi:10.3389/fcimb.2025.1684624)

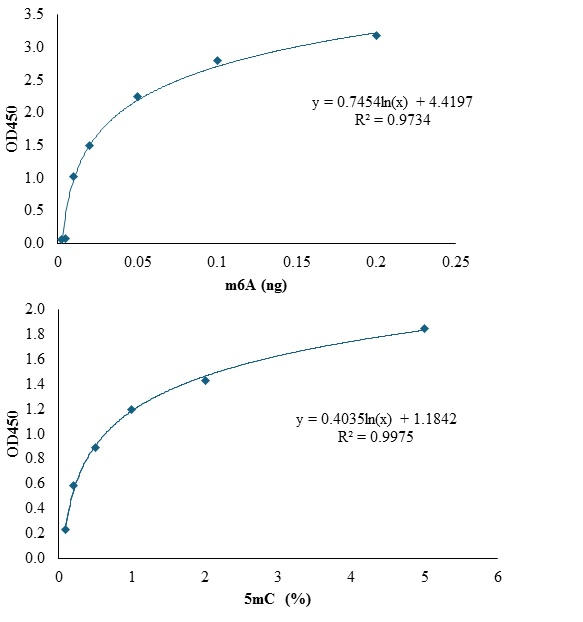

Supplement: Supplementary Figure 1 — Representative standard curves generated with m6A and m5C standard controls. [file Image1.tiff]
