# Supplementary material for: Antimicrobial effects of essential oil from Origanum vulgare in combination with conventional antibiotics against Staphylococcus aureus
Source: Front Cell Infect Microbiol. 2025 Oct 23;15:1684624. doi: 10.3389/fcimb.2025.1684624 (PMC12588934; doi:10.3389/fcimb.2025.1684624)
Supplement: Supplementary file 2 [file DataSheet1.pdf]

Table S1. Characterization of the Essential Oils analyzed (presence ≥ 1%).

Essential Oil	Family	Extraction method	Chemical Composition	%	Retention Index	Cultivation area	Time of Collection	Batch N.
Clinopodium nepeta	*Lamiaceae*	Hydro distillation from fresh collected material	piperitone oxide	34,28	16,14	Briatico, Province of Vibo Valentia, Calabria Region, Southern Italy Farm Crudo Michele	July 2021	20210715
			piperitenone oxide	18,23	19,72			
			(+)-limonene	15,8	8,49			
			(+)-pulegone	13,75	15,6			
			menthone	8,32	12,77			
			isolegylacetate	3,64	17,92			
			1-terpine-4-ol	1,4	13,56			
			(+)-neomenthol	1,37	13,25			
			β-pinene	1,22	6,92			
Citrus bergamia	*Rutaceae*	Mechanical extraction through industrial cold expression process from fresh fruit	(+)-limonene	15,89	34.28	Bovalino, Province of Reggio Calabria, Calabria Region, Southern Italy Farm Frammartino	December 2021	20211221
			lynalyl acetate	10,78	11.54			
			(+)-linalool	9,38	6.79			
			α -terpinene	8,44	38.88			
			β-pinene	6,87	5.49			
			α -pinene	5,64	1,22			
Citrus limon (L.)	*Rutaceae*	Hydro distillation from fresh collected material	(+)-limonene	14,13	3.01	Bovalino, Province of Reggio Calabria, Calabria Region, Southern Italy Farm Frammartino	November 2021	2021118
			α -terpinene	13,59	1.26			
			β-pinene	10,28	1.67			
			α -terpineol	9,36	11.91			
			α -terpinolene	8,42	74.41			
			1-Terpine-4-ol	6,84	4.34			
Citrus reticulata	*Rutaceae*	Hydro distillation from fresh collected material	(+)-sabinene	12,6	1.44	Briatico, Province of Vibo Valentia, Calabria Region, Southern Italy Farm Crudo Michele	February 2021	20210215
			(+)-linalool	10,76	18.27			
			α -phellandrene	10,27	1.37			
			β -cis-ocimene	9,36	1.14			
			(+)-limonene	8,96	6.45			
			β -myrcene	8,42	5.04			

			β -pinene	7,76	6.54			
			α -pinene	7,16	2.37			
			β -citronellal	6,86	2.35			
			α -terpinolene	6,72	50.91			
			α -terpinene	5,63	1.93			
Foeniculum vulgare subsp. *piperitum*	*Apiaceae*	Hydro distillation from fresh collected material	estragole	17,21	14.54	Briatico, Province of Vibo Valentia, Calabria Region, Southern Italy Farm Crudo Michele	September 2021	20210918
			α -pinene	14,19	45.33			
			anethal	10,41	11.24			
			fenchone	8,42	8.49			
			α -limonene	7,72	2.51			
			α -phellandrene	7,18	1.05			
			β -pinene	6,86	1.65			
			β -myrcene	5,63	14.71			
Laurus nobilis L.	*Lauraceae*	Hydro distillation from fresh collected material	eucalyptol	21,07	1,51	Briatico, Province of Vibo Valentia, Calabria Region, Southern Italy Farm Crudo Michele	April 2021	20210413
			(+)- sabinene	19,18	6,48			
			(+)-linalool	13,6	1,29			
			terpinyl acetate	10,79	7,38			
			α -pinene	8,56	56,61			
			methyleugenol	6,73	15,74			
			1-terpine-4-ol	5,64	5,65			
Myrtus communis L.	*Myrtaceae*	Hydro distillation from fresh collected material	eucalyptol	20,27	1,88	Briatico, Province of Vibo Valentia, Calabria Region, Southern Italy Farm Crudo Michele	May 2021	20210521
			(-)-myrtenylacetate	18,43	17,04			
			α -pinene	15,92	3,88			
			(+)-limonene	14,16	2,1			
			(+)-linalool	10,82	10,43			
			lynalyl acetate	10,33	1,1			
			geraniol acetate	9,44	1,09			
			α -terpineol	9,04	1,58			
			β -ocimene	8,59	33,04			

			α -phellandrene<br>o-cymene<br>terpinolene<br>terpinene	8,49<br>8,35<br>7,79<br>5,69	10,81<br>1,41<br>1,41<br>12,33			
Origanum vulgare L. subsp. *viridulum*	*Lamiaceae*	Hydro distillation from fresh collected material	p-thymol<br>terpinene<br>p-cymene<br>β -caryophyllene<br>β -myrcene<br>carvacrol<br>terpinolene<br>α -thujene (origanene)<br>α -pinene	20,48<br>16,77<br>16,42<br>8,63<br>7,61<br>7,36<br>6,55<br>5,15<br>4,96	4,88<br>47,31<br>3,52<br>18,52<br>11,78<br>3,18<br>3,76<br>1,23<br>2,73	Briatico, Province of Vibo Valentia, Calabria Region, Southern Italy Farm Crudo Michele	July 2021	20210720
Salvia officinalis L.	*Lamiaceae*	Hydro distillation from fresh collected material	eucalyptol<br>(-)-α -thujone<br>β -pinene<br>(-)-camphor<br>α -humulene<br>(-)-β -thujone<br>α -pinene<br>(-)-β - caryophyllene<br>β -myrcene<br>C<br>camphene<br>(+)- sabinene	22,6<br>21,5<br>12,45<br>11,44<br>11,07<br>8,58<br>7,23<br>6,9<br>6,75<br>6,12<br><br>5,67	5,54<br>2,85<br>9,59<br>4,35<br>24,14<br>23,7<br>2,26<br>15,1<br>1,13<br>1,88<br><br>3,99	Briatico, Province of Vibo Valentia, Calabria Region, Southern Italy Farm Crudo Michele	May 2021	20210504
Salvia rosmarinus	*Lamiaceae*	Hydro distillation from fresh collected material	eucalyptol<br>α -pinene<br>β -pinene<br>camphene<br>(-)-camphor	21,52<br>13,31<br>12,45<br>8,56<br>7,21	1,17<br>2,28<br>3,66<br>49,29<br>1,79	Briatico, Province of Vibo Valentia, Calabria Region, Southern Italy Farm Crudo Michele	April 2021	20210421

			isoborneol<br>β -myrcene<br>(-)-β -caryophyllene	6,89<br>6,1<br>5,66	9,26<br>6,7<br>22,84			
