# Supplementary material for: Antimicrobial effects of essential oil from Origanum vulgare in combination with conventional antibiotics against Staphylococcus aureus
Source: Front Cell Infect Microbiol. 2025 Oct 23;15:1684624. doi: 10.3389/fcimb.2025.1684624 (PMC12588934; doi:10.3389/fcimb.2025.1684624)
Supplement: Supplementary file 3 [file DataSheet2.pdf]

Table 2. Characteristics of the plant families from which the Essential Oils analyzed in the study were extracted

Family	Habit (Life-form)	Leaves	Inflorescence & Flowers	Fruit/Seeds
**Lamiaceae**	Herbs, shrubs, trees or lianas; often aromatic	Opposite or whorled; simple or compound; no stipules; glandular hairs common	Zygomorphic, bilabiate corolla; verticillasters or cymes; 2–4 stamens	Schizocarp of 4 nutlets or drupe; often enclosed in persistent calyx
**Rutaceae**	Trees, shrubs; some herbs; often aromatic with gland-dotted leaves	Usually opposite, compound, no stipules; pellucid oil glands (aromatic)	Hermaphrodites, actinomorphic, typically 4–5 merous; 8–10 stamens; separated carpels	Variable: berries, drupes, hesperidia, samaras, capsules, follicles
**Apiaceae**	Mostly annual, biennial, or perennial herbs; some woody shrubs/trees	Usually alternate, strongly dissected; sheathing petioles; no stipules; aromatic	Umbels (simple/compound); small actinomorphic flowers; 5-merous; inferior ovary	Dry schizocarp splitting into two mericarps with oil canals (vittae)
**Myrtaceae**	Trees, shrubs, subshrubs; evergreen, often aromatic	Simple, opposite or alternate; entire margins; oil glands visible; aromatic	Hermaphrodites, actinomorphic; many stamens; hypanthium present; ovary inferior or semi-inferior	Mostly fleshy berries; also capsules; seeds often without endosperm
**Lauraceae**	Mostly evergreen trees or shrubs; rarely parasitic/climbing; aromatic	Alternate or whorled, simple, leathery; evergreen; with oil cavities	Small, grouped in inflorescences (panicles or umbels); 3–12 stamens with nectar appendages	Fleshy drupe or berry, often with cupule at base; single-seeded
