# Supplementary material for: Antimicrobial effects of essential oil from Origanum vulgare in combination with conventional antibiotics against Staphylococcus aureus
Source: Front Cell Infect Microbiol. 2025 Oct 23;15:1684624. doi: 10.3389/fcimb.2025.1684624 (PMC12588934; doi:10.3389/fcimb.2025.1684624)
Supplement: Supplementary file 4 [file DataSheet3.pdf]

Table S3. OD_{600} average values of three independent experiments obtained by the checkerboard assays.

ANTIBIOTICS (μg/mL)		OEO (mg/mL)															
		0.000		0.010		0.020		0.040		0.080		0.160		0.320		0.640	
		Mean	SEM	Mean	SEM	Mean	SEM	Mean	SEM	Mean	SEM	Mean	SEM	Mean	SEM	Mean	SEM
**Ampicillin**	**0.000**	0.323	0.008	0.294	0.018	0.291	0.016	0.286	0.016	0.267	0.004	0.177	0.014	0.000	0.000	0.000	0.000
	**0.016**	0.273	0.004	0.114	0.009	0.148	0.003	0.207	0.003	0.233	0.003	0.019	0.013	0.001	0.000	0.000	0.000
	**0.031**	0.251	0.025	0.131	0.014	0.166	0.011	0.151	0.018	0.161	0.019	0.004	0.012	0.001	0.000	0.000	0.000
	**0.063**	0.201	0.035	0.127	0.016	0.157	0.008	0.131	0.008	0.153	0.009	0.001	0.015	0.001	0.000	0.001	0.000
	**0.125**	0.112	0.031	0.109	0.019	0.144	0.003	0.125	0.009	0.103	0.011	0.001	0.013	0.001	0.000	0.000	0.000
	**0.250**	0.067	0.025	0.087	0.003	0.116	0.000	0.116	0.003	0.081	0.003	0.000	0.003	0.001	0.000	0.000	0.000
	**0.500**	0.005	0.000	0.005	0.000	0.005	0.000	0.005	0.000	0.005	0.000	0.005	0.000	0.005	0.000	0.005	0.000
	**1.000**	0.005	0.000	0.005	0.000	0.005	0.000	0.005	0.000	0.005	0.000	0.005	0.000	0.005	0.000	0.005	0.000
**Ciprofloxaci n**	**0.000**	0.328	0.073	0.322	0.075	0.326	0.106	0.333	0.085	0.322	0.097	0.326	0.058	0.328	0.102	0.005	0.000
	**0.004**	0.282	0.038	0.286	0.019	0.283	0.085	0.282	0.093	0.281	0.104	0.282	0.036	0.286	0.100	0.005	0.000
	**0.008**	0.238	0.082	0.227	0.047	0.226	0.034	0.237	0.105	0.233	0.057	0.229	0.081	0.229	0.027	0.005	0.000
	**0.016**	0.211	0.034	0.205	0.022	0.222	0.093	0.209	0.099	0.212	0.000	0.200	0.064	0.205	0.000	0.005	0.000
	**0.031**	0.162	0.006	0.168	0.024	0.154	0.046	0.169	0.027	0.146	0.067	0.169	0.005	0.169	0.039	0.005	0.000
	**0.063**	0.078	0.020	0.088	0.002	0.000	0.070	0.085	0.001	0.082	0.001	0.085	0.002	0.079	0.001	0.005	0.000
	**0.125**	0.005	0.000	0.005	0.000	0.005	0.000	0.005	0.000	0.005	0.000	0.005	0.000	0.005	0.000	0.005	0.000
	**0.250**	0.005	0.000	0.005	0.000	0.005	0.000	0.005	0.000	0.005	0.000	0.005	0.000	0.005	0.000	0.005	0.000
**Erytromycin**	**0.000**	0.401	0.050	0.414	0.009	0.402	0.015	0.405	0.006	0.429	0.024	0.381	0.011	0.050	0.000	0.050	0.000
	**0.008**	0.399	0.045	0.387	0.005	0.402	0.036	0.400	0.012	0.389	0.018	0.372	0.010	0.050	0.000	0.050	0.000
	**0.016**	0.392	0.025	0.383	0.029	0.435	0.070	0.382	0.022	0.379	0.024	0.393	0.024	0.050	0.000	0.050	0.000
	**0.031**	0.350	0.036	0.385	0.017	0.408	0.026	0.365	0.014	0.364	0.042	0.351	0.011	0.050	0.000	0.050	0.000
	**0.063**	0.311	0.087	0.337	0.012	0.333	0.012	0.324	0.006	0.332	0.006	0.326	0.018	0.050	0.000	0.050	0.000
	**0.125**	0.220	0.033	0.245	0.028	0.242	0.051	0.232	0.011	0.240	0.003	0.235	0.012	0.050	0.000	0.050	0.000
	**0.250**	0.050	0.000	0.050	0.000	0.050	0.000	0.050	0.000	0.050	0.000	0.050	0.000	0.050	0.000	0.050	0.000
	**0.500**	0.050	0.000	0.050	0.000	0.050	0.000	0.050	0.000	0.050	0.000	0.050	0.000	0.050	0.000	0.050	0.000
**Gentamicin**	**0.000**	0.332	0.007	0.291	0.039	0.263	0.051	0.161	0.031	0.042	0.086	0.033	0.010	0.050	0.000	0.050	0.000
	**0.063**	0.330	0.023	0.145	0.023	0.151	0.059	0.152	0.035	0.138	0.005	0.119	0.045	0.050	0.000	0.050	0.000

	**0.125**	0.326	0.046	0.143	0.073	0.146	0.022	0.134	0.104	0.143	0.061	0.060	0.002	0.050	0.000	0.050	0.000
	**0.250**	0.321	0.094	0.164	0.064	0.169	0.007	0.218	0.089	0.011	0.085	0.050	0.000	0.050	0.000	0.050	0.000
	**0.500**	0.302	0.031	0.173	0.084	0.133	0.050	0.050	0.000	0.050	0.000	0.050	0.000	0.050	0.000	0.050	0.000
	**1.000**	0.209	0.035	0.050	0.000	0.050	0.000	0.050	0.000	0.050	0.000	0.050	0.000	0.050	0.000	0.050	0.000
	**2.000**	0.050	0.000	0.050	0.000	0.050	0.000	0.050	0.000	0.050	0.000	0.050	0.000	0.050	0.000	0.050	0.000
	**4.000**	0.050	0.000	0.050	0.000	0.050	0.000	0.050	0.000	0.050	0.000	0.050	0.000	0.050	0.000	0.050	0.000
**Levofloxacin**	**0.000**	0.309	0.016	0.298	0.078	0.305	0.057	0.307	0.033	0.302	0.057	0.304	0.087	0.050	0.000	0.050	0.000
	**0.008**	0.294	0.091	0.289	0.080	0.293	0.077	0.296	0.003	0.301	0.026	0.299	0.015	0.050	0.000	0.050	0.000
	**0.016**	0.260	0.037	0.254	0.005	0.259	0.078	0.261	0.040	0.266	0.059	0.264	0.029	0.050	0.000	0.050	0.000
	**0.031**	0.256	0.103	0.256	0.045	0.238	0.023	0.274	0.052	0.265	0.020	0.300	0.014	0.050	0.000	0.050	0.000
	**0.063**	0.222	0.062	0.217	0.066	0.221	0.075	0.224	0.001	0.229	0.077	0.227	0.003	0.050	0.000	0.050	0.000
	**0.125**	0.154	0.009	0.133	0.096	0.138	0.058	0.147	0.033	0.130	0.084	0.128	0.005	0.050	0.000	0.050	0.000
	**0.250**	0.050	0.000	0.050	0.000	0.050	0.000	0.050	0.000	0.050	0.000	0.050	0.000	0.050	0.000	0.050	0.000
	**0.500**	0.050	0.000	0.050	0.000	0.050	0.000	0.050	0.000	0.050	0.000	0.050	0.000	0.050	0.000	0.050	0.000
**Tetracycline**	**0.000**	0.309	0.010	0.307	0.010	0.279	0.035	0.275	0.052	0.257	0.000	0.170	0.000	0.050	0.000	0.050	0.000
	**0.031**	0.275	0.001	0.172	0.093	0.155	0.075	0.154	0.029	0.097	0.024	0.002	0.000	0.050	0.000	0.050	0.000
	**0.063**	0.255	0.058	0.163	0.084	0.141	0.090	0.132	0.027	0.095	0.062	0.002	0.000	0.050	0.000	0.050	0.000
	**0.125**	0.153	0.048	0.128	0.010	0.131	0.035	0.093	0.052	0.051	0.004	0.002	0.000	0.050	0.000	0.050	0.000
	**0.250**	0.034	0.020	0.039	0.025	0.037	0.023	0.028	0.008	0.025	0.002	0.002	0.000	0.050	0.000	0.050	0.000
	**0.500**	0.026	0.015	0.030	0.003	0.028	0.002	0.010	0.002	0.007	0.070	0.003	0.000	0.050	0.000	0.050	0.000
	**1.000**	0.050	0.000	0.050	0.000	0.050	0.000	0.050	0.000	0.050	0.000	0.050	0.000	0.050	0.000	0.050	0.000
	**2.000**	0.050	0.000	0.050	0.000	0.050	0.000	0.050	0.000	0.050	0.000	0.050	0.000	0.050	0.000	0.050	0.000
**Tobramycin**	**0.000**	0.346	0.010	0.315	0.018	0.311	0.014	0.306	0.013	0.286	0.004	0.189	0.071	0.050	0.000	0.050	0.000
	**0.063**	0.350	0.014	0.346	0.012	0.353	0.014	0.354	0.014	0.347	0.013	0.301	0.009	0.050	0.000	0.050	0.000
	**0.125**	0.356	0.011	0.364	0.011	0.360	0.019	0.360	0.020	0.348	0.010	0.016	0.022	0.050	0.000	0.050	0.000
	**0.250**	0.325	0.020	0.303	0.011	0.314	0.020	0.306	0.006	0.044	0.035	0.010	0.018	0.050	0.000	0.050	0.000
	**0.500**	0.310	0.014	0.223	0.005	0.209	0.132	0.191	0.034	0.006	0.004	0.008	0.007	0.050	0.000	0.050	0.000
	**1.000**	0.206	0.021	0.118	0.013	0.077	0.013	0.003	0.011	0.003	0.009	0.006	0.014	0.050	0.000	0.050	0.000
	**2.000**	0.050	0.000	0.050	0.000	0.050	0.000	0.050	0.000	0.050	0.000	0.050	0.000	0.050	0.000	0.050	0.000
	**4.000**	0.050	0.000	0.050	0.000	0.050	0.000	0.050	0.000	0.050	0.000	0.050	0.000	0.050	0.000	0.050	0.000
