# Supplementary material for: Antimicrobial effects of essential oil from Origanum vulgare in combination with conventional antibiotics against Staphylococcus aureus
Source: Front Cell Infect Microbiol. 2025 Oct 23;15:1684624. doi: 10.3389/fcimb.2025.1684624 (PMC12588934; doi:10.3389/fcimb.2025.1684624)
Supplement: Supplementary file 5 [file DataSheet4.pdf]

Table S4. ZIP scores of the interactions between OEO and antibiotics at all the concentrations analyzed. FIC_I is reported only for those ZIP values equal or higher of 10 (bold). ND: Not Determined

**OEO concentration (mg/mL)**	**Ampicillin concentration (μg/mL)**	**ZIP Score**	**FIC_I**
0.01	0.0160	**26.710**	0.064
0.01	0.0310	**22.750**	0.094
0.01	0.0630	**10.540**	0.158
0.01	0.1250	3.720	ND
0.01	0.2500	-3.060	ND
0.01	0.5000	-0.550	ND
0.02	0.0160	**23.750**	0.096
0.02	0.0310	**20.130**	0.126
0.02	0.0630	8.080	ND
0.02	0.1250	-0.690	ND
0.02	0.2500	-6.150	ND
0.02	0.5000	-1.940	ND
0.04	0.0160	**27.150**	0.160
0.04	0.0310	**23.650**	0.190
0.04	0.0630	**11.820**	0.254
0.04	0.1250	-0.780	ND
0.04	0.2500	-5.820	ND
0.04	0.5000	-1.060	ND
0.08	0.0160	**26.950**	0.288
0.08	0.0310	**24.520**	0.318
0.08	0.0630	**15.220**	0.382
0.08	0.1250	0.980	ND
0.08	0.2500	-3.750	ND
0.08	0.5000	1.010	ND
0.16	0.0160	**31.780**	0.545
0.16	0.0310	**31.090**	0.575
0.16	0.0630	**23.220**	0.639
0.16	0.1250	**19.820**	0.763
0.16	0.2500	**10.061**	1.013
0.16	0.5000	2.680	ND
0.312	0.0160	-3.060	ND
0.312	0.0310	-1.170	ND
0.312	0.0630	-0.610	ND
0.312	0.1250	1.040	ND
0.312	0.2500	0.480	ND
0.312	0.5000	0.040	ND

**OEO concentration (mg/mL)**	**Gentamicin concentration (µg/mL)**	**ZIP Score**	**FIC_I**
0.01	0.0625	**34.940**	0.063
0.01	0.1250	**24.840**	0.095
0.01	0.2500	0.900	ND
0.01	0.5000	-5.660	ND
0.01	1.0000	-11.500	ND
0.01	2.0000	-4.870	ND
0.02	0.0625	**40.500**	0.095
0.02	0.1250	**30.800**	0.127
0.02	0.2500	3.340	ND
0.02	0.5000	-3.000	ND
0.02	1.0000	-6.690	ND
0.02	2.0000	-1.860	ND
0.04	0.0625	**39.120**	0.159
0.04	0.1250	**32.490**	0.191
0.04	0.2500	3.520	ND
0.04	0.5000	4.250	ND
0.04	1.0000	-0.028	ND
0.04	2.0000	1.500	ND
0.08	0.0625	**45.730**	0.288
0.08	0.1250	**46.900**	0.319
0.08	0.2500	**39.770**	0.381
0.08	0.5000	**15.460**	0.506
0.08	1.0000	5.140	ND
0.08	2.0000	2.480	ND
0.16	0.0625	**47.990**	0.544
0.16	0.1250	**40.670**	0.575
0.16	0.2500	**29.140**	0.638
0.16	0.5000	**10.550**	0.763
0.16	1.0000	2.940	ND
0.16	2.0000	0.960	ND
0.312	0.0625	-2.380	ND
0.312	0.1250	-1.530	ND
0.312	0.2500	0.610	ND
0.312	0.5000	0.130	ND
0.312	1.0000	-0.084	ND
0.312	2.0000	-0.098	ND

**OEO concentration (mg/mL)**	**Tetracycline concentration (µg/mL)**	**ZIP Score**	**FIC_I**
0.01	0.0313	**25.460**	0.063
0.01	0.0625	**23.830**	0.095
0.01	0.1250	4.570	ND
0.01	0.2500	-2.260	ND
0.01	0.5000	-5.840	ND
0.01	1.0000	-0.065	ND
0.02	0.0313	**33.930**	0.095
0.02	0.0625	**30.920**	0.127
0.02	0.1250	8.340	ND
0.02	0.2500	-1.910	ND
0.02	0.5000	-5.880	ND
0.02	1.0000	-0.170	ND
0.04	0.0313	**40.860**	0.159
0.04	0.0625	**38.800**	0.191
0.04	0.1250	**17.100**	0.253
0.04	0.2500	1.290	ND
0.04	0.5000	-1.130	ND
0.04	1.0000	0.830	ND
0.08	0.0313	**53.500**	0.288
0.08	0.0625	**49.030**	0.319
0.08	0.1250	**27.150**	0.381
0.08	0.2500	4.480	ND
0.08	0.5000	0.520	ND
0.08	1.0000	0.630	ND
0.16	0.0313	**42.410**	0.544
0.16	0.0625	**36.410**	0.575
0.16	0.1250	**22.730**	0.638
0.16	0.2500	6.850	ND
0.16	0.5000	1.130	ND
0.16	1.0000	0.220	ND
0.312	0.0313	2.400	ND
0.312	0.0625	1.980	ND
0.312	0.1250	1.970	ND
0.312	0.2500	0.540	ND
0.312	0.5000	-0.380	ND
0.312	1.0000	-0.200	ND

**OEO concentration (mg/mL)**	**Tobramycin concentration (μg/mL)**	**ZIP Score**	**FIC_I**
0.01	0.0625	-6.510	ND
0.01	0.1250	-7.160	ND
0.01	0.2500	-0.810	ND
0.01	0.5000	**14.530**	0.282
0.01	1.0000	**19.880**	0.532
0.01	2.0000	0.690	ND
0.02	0.0625	-5.990	ND
0.02	0.1250	-6.760	ND
0.02	0.2500	-0.530	ND
0.02	0.5000	**24.850**	0.314
0.02	1.0000	**34.390**	0.564
0.02	2.0000	1.830	ND
0.04	0.0625	-5.360	ND
0.04	0.1250	-6.340	ND
0.04	0.2500	0.980	ND
0.04	0.5000	**40.380**	0.378
0.04	1.0000	**49.560**	0.628
0.04	2.0000	2.820	ND
0.08	0.0625	-4.790	ND
0.08	0.1250	-4.470	ND
0.08	0.2500	**74.130**	0.381
0.08	0.5000	**75.700**	0.506
0.08	1.0000	**50.490**	0.756
0.08	2.0000	2.460	ND
0.16	0.0625	-8.500	ND
0.16	0.1250	**47.710**	0.575
0.16	0.2500	**51.040**	0.638
0.16	0.5000	**48.020**	0.763
0.16	1.0000	**30.280**	1.013
0.16	2.0000	0.970	ND
0.312	0.0625	3.100	ND
0.312	0.1250	3.120	ND
0.312	0.2500	2.660	ND
0.312	0.5000	3.860	ND
0.312	1.0000	2.040	ND
0.312	2.0000	0.100	ND

**OEO concentration (mg/mL)**	**Ciprofloxacin concentration (µg/mL)**	**ZIP Score**
0.01	0.0039	0.670
0.01	0.0078	2.610
0.01	0.0156	-0.810
0.01	0.0312	-3.240
0.01	0.0625	-0.670
0.01	0.1250	3.050
0.02	0.0039	0.160
0.02	0.0078	2.300
0.02	0.0156	-0.820
0.02	0.0312	-2.920
0.02	0.0625	-0.200
0.02	0.1250	3.380
0.04	0.0039	-0.140
0.04	0.0078	2.110
0.04	0.0156	-1.060
0.04	0.0312	-3.360
0.04	0.0625	-0.740
0.04	0.1250	3.040
0.08	0.0039	0.300
0.08	0.0078	2.520
0.08	0.0156	-0.350
0.08	0.0312	-2.160
0.08	0.0625	0.600
0.08	0.1250	3.780
0.16	0.0039	0.800
0.16	0.0078	2.980
0.16	0.0156	-0.390
0.16	0.0312	-2.940
0.16	0.0625	-0.530
0.16	0.1250	3.060
0.312	0.0039	0.240
0.312	0.0078	3.930
0.312	0.0156	0.470
0.312	0.0312	-2.840
0.312	0.0625	-0.210
0.312	0.1250	3.350

**OEO concentration (mg/mL)**	**Erytromycin concentration (µg/mL)**	**ZIP Score**
0.01	7.81	-2.780
0.01	15.63	-2.120
0.01	31.25	1.740
0.01	62.50	0.480
0.01	125.00	-5.450
0.01	250.00	4.300
0.02	7.81	-1.310
0.02	15.63	-0.750
0.02	31.25	2.590
0.02	62.50	-0.480
0.02	125.00	-6.250
0.02	250.00	5.580
0.04	7.81	-1.550
0.04	15.63	-0.770
0.04	31.25	3.490
0.04	62.50	3.150
0.04	125.00	-3.610
0.04	250.00	4.010
0.08	7.81	-4.160
0.08	15.63	-2.610
0.08	31.25	3.490
0.08	62.50	5.450
0.08	125.00	-3.330
0.08	250.00	1.820
0.16	7.81	-2.280
0.16	15.63	-5.120
0.16	31.25	-1.730
0.16	62.50	-4.880
0.16	125.00	-9.430
0.16	250.00	5.410
0.312	7.81	0.015
0.312	15.63	0.031
0.312	31.25	-0.013
0.312	62.50	0.000
0.312	125.00	-0.019
0.312	250.00	0.003

**OEO concentration (mg/mL)**	**Levofloxacin concentration (μg/mL)**	**ZIP Score**
0.01	0.0078	-4.740
0.01	0.0156	1.450
0.01	0.0313	2.490
0.01	0.0625	4.240
0.01	0.1250	-1.830
0.01	0.2500	4.010
0.02	0.0078	-5.040
0.02	0.0156	1.940
0.02	0.0313	3.890
0.02	0.0625	5.560
0.02	0.1250	-2.280
0.02	0.2500	2.820
0.04	0.0078	-6.360
0.04	0.0156	-0.410
0.04	0.0313	0.220
0.04	0.0625	1.830
0.04	0.1250	-3.270
0.04	0.2500	3.790
0.08	0.0078	-5.820
0.08	0.0156	-0.150
0.08	0.0313	0.076
0.08	0.0625	1.840
0.08	0.1250	-1.940
0.08	0.2500	4.950
0.16	0.0078	-6.350
0.16	0.0156	-1.050
0.16	0.0313	-1.690
0.16	0.0625	-0.330
0.16	0.1250	2.640
0.16	0.2500	5.920
0.312	0.0078	-0.001
0.312	0.0156	-0.022
0.312	0.0313	-0.041
0.312	0.0625	-0.027
0.312	0.1250	-0.150
0.312	0.2500	0.000
