# Supplementary material for: Antimicrobial effects of essential oil from Origanum vulgare in combination with conventional antibiotics against Staphylococcus aureus
Source: Front Cell Infect Microbiol. 2025 Oct 23;15:1684624. doi: 10.3389/fcimb.2025.1684624 (PMC12588934; doi:10.3389/fcimb.2025.1684624)
Supplement: Supplementary file 6 [file DataSheet5.pdf]

Table S5. OD_{450} average values of three independent experiments obtained by crystal violet method for the determination of biofilm. OEO: Essential Oil from *Origanum vulgare*; AMP: ampicillin, GEN: gentamicin, TET: tetracycline, TOB: tobramycin.

	**OD_{570}**	
	**Mean**	**SEM**
AMP	0.410	0.032
AMP+ OEO	0.194	0.028
GEN	0.550	0.098
GEN + OEO	0.150	0.017
TET	0.348	0.048
TET + OEO	0.102	0.002
TOB	0.436	0.070
TOB + OEO	0.041	0.010
