# Supplementary material for: Antimicrobial effects of essential oil from Origanum vulgare in combination with conventional antibiotics against Staphylococcus aureus
Source: Front Cell Infect Microbiol. 2025 Oct 23;15:1684624. doi: 10.3389/fcimb.2025.1684624 (PMC12588934; doi:10.3389/fcimb.2025.1684624)
Supplement: Supplementary file 7 [file DataSheet6.pdf]

Table S6. C_t average values of three independent experiments obtained by quantitative real-time PCR reactions. OEO: Essential Oil from *Origanum vulgare*; AMP: ampicillin, GEN: gentamicin, TET: tetracycline, TOB: tobramycin.

	agrA		***hld***		***RNAIII***		***rot***		***16S rRNA***	
	**Mean**	**ESM**	**Mean**	**ESM**	**Mean**	**ESM**	**Mean**	**ESM**	**Mean**	**ESM**
**AMP**	15.33	0.04	15.05	0.04	16.33	0.01	16.85	0.01	10.30	0.09
**AMP+ OEO**	15.31	0.07	15.63	0.10	16.21	0.02	18.07	0.10	10.19	0.05
**GEN**	15.28	0.09	14.94	0.04	16.26	0.09	16.51	0.07	10.27	0.06
**GEN + OEO**	15.50	0.04	15.36	0.03	16.77	0.07	17.63	0.08	10.28	0.08
**TET**	15.25	0.04	15.09	0.02	16.31	0.09	16.37	0.02	10.89	0.1
**TET + OEO**	15.81	0.01	16.27	0.04	17.10	0.10	17.75	0.01	10.78	0.03
**TOB**	15.29	0.08	15.15	0.01	16.19	0.07	17.10	0.09	11.10	0.03
**TOB + OEO**	15.49	0.07	15.44	0.01	16.32	0.10	18.15	0.10	11.21	0.02
