# Supplementary material for: Antimicrobial effects of essential oil from Origanum vulgare in combination with conventional antibiotics against Staphylococcus aureus
Source: Front Cell Infect Microbiol. 2025 Oct 23;15:1684624. doi: 10.3389/fcimb.2025.1684624 (PMC12588934; doi:10.3389/fcimb.2025.1684624)
Supplement: Supplementary file 8 [file DataSheet7.pdf]

Table S7. OD_{450} average values of three independent experiments obtained by ELISA assay for the quantification of global adenine (**A**) and cytosine methylation (**B**). OEO: Essential Oil from *Origanum vulgare*; AMP: ampicillin, GEN: gentamicin, TET: tetracycline, TOB: tobramycin.

A

	**OD_{450}**	
	**Mean**	**SEM**
**AMP**	1.227	0.047
**AMP+ OEO**	0.558	0.069
**GEN**	1.233	0.094
**GEN + OEO**	0.906	0.099
**TET**	1.082	0.074
**TET + OEO**	0.498	0.085
**TOB**	1.419	0.055
**TOB + OEO**	0.924	0.082

B

	**OD_{450}**	
	**Mean**	**SEM**
**AMP**	0.108	0.032
**AMP+ OEO**	0.549	0.028
**GEN**	0.119	0.080
**GEN + OEO**	0.591	0.017
**TET**	0.107	0.069
**TET + OEO**	0.426	0.071
**TOB**	0.113	0.087
**TOB + OEO**	0.090	0.010
